# Hepatocyte Nuclear Factor 4 Alpha Polymorphisms and the Metabolic Syndrome in French-Canadian Youth

**DOI:** 10.1371/journal.pone.0117238

**Published:** 2015-02-11

**Authors:** Valérie Marcil, Devendra Amre, Ernest G. Seidman, François Boudreau, Fernand P. Gendron, Daniel Ménard, Jean François Beaulieu, Daniel Sinnett, Marie Lambert, Emile Levy

**Affiliations:** 1 Research Institute, McGill University, Montreal, Quebec, Canada, H3G 1A4; 2 Department of Pediatrics, CHU-Sainte-Justine, Université de Montréal, Montreal, Quebec, Canada, H3T 1C5; 3 Canadian Institutes for Health Research Team on the Digestive Epithelium, Department of Anatomy and Cellular Biology, Faculty of Medicine and Health Sciences, Université de Sherbrooke, Sherbrooke, Quebec, Canada, J1H 5N4; 4 Department of Nutrition, CHU-Sainte-Justine, Université de Montréal, Montreal, Quebec, Canada, H3T 1C5; Univeristy of California Riverside, UNITED STATES

## Abstract

**Objectives:**

Hepatocyte nuclear factor 4 alpha (HNF4α) is a transcription factor involved in the regulation of serum glucose and lipid levels. Several *HNF4A* gene variants have been associated with the risk of developing type 2 diabetes mellitus. However, no study has yet explored its association with insulin resistance and the cardiometabolic risk in children. We aimed to investigate the relationship between *HNF4A* genetic variants and the presence of metabolic syndrome (MetS) and metabolic parameters in a pediatric population.

**Design and Methods:**

Our study included 1,749 French-Canadians aged 9, 13 and 16 years and evaluated 24 *HNF4A* polymorphisms that were previously identified by sequencing.

**Results:**

Analyses revealed that, after correction for multiple testing, one SNP (rs736824; *P*<0.022) and two haplotypes (P1 promoter haplotype rs6130608-rs2425637; *P*<0.032 and intronic haplotype rs736824-rs745975-rs3212183; *P*<0.025) were associated with the risk of MetS. Additionally, a significant association was found between rs3212172 and apolipoprotein B levels (coefficient: -0.14 ± 0.05; *P*<0.022). These polymorphisms are located in *HNF4A* P1 promoter or in intronic regions.

**Conclusions:**

Our study demonstrates that HNF4α genetic variants are associated with the MetS and metabolic parameters in French Canadian children and adolescents. This study, the first exploring the relation between *HNF4A* genetic variants and MetS and metabolic variables in a pediatric cohort, suggests that HNF4α could represent an early marker for the risk of developing type 2 diabetes mellitus.

## INTRODUCTION

The constantly rising prevalence of childhood obesity is becoming one of the most alarming public health problems worldwide. In parallel, there has been a significant increase in the number of children and adolescents with clinical signs of insulin resistance (IR) and prediabetes [1], which are likely to progress to type 2 diabetes mellitus (T2D) and early atherosclerosis [2]. Development of T2D in young people is of particular concern because complications are common and appear early in the disease [3,4]. Consequently, the identification of early markers and genetic risk factors for IR and T2D are becoming important tools for the management and the prevention of long-term cardiometabolic consequences.

The hepatocyte nuclear factor 4 alpha (HNF4α; *HNF4A*) is a member of the nuclear receptor superfamily of ligand-dependant transcription factors [5] and is mainly expressed in liver, intestine, pancreatic islets and kidney [5]. It influences lipid transport and metabolism [6–8] and is essential to hepatocyte differentiation and liver function [9]. Moreover, HNF4α maintains glucose homeostasis by regulating gene expression in pancreatic β cells [10–12] and gluconeogenesis in the liver [13,14].

The *HNF4A* gene is composed of thirteen exons and two promoters that drive the expression of many splice variants (isoforms) [15], for which 6 of the 9 splice variants appear to yield to full length transcripts [16,17]. The transcription of three of these isoforms is driven by an alternate promoter known as P2, which is located 45.6 kb upstream P1 promoter [18,19]. P2-driven transcripts have been described as the predominant splice variant in pancreatic β-cells [18–21], while the P1 promoter appears to be mainly active in liver cells [19,22,23].

Mutations in both the coding and regulatory regions of HNF4α have been associated with maturity-onset diabetes of the young (MODY)-1, a dominantly inherited, atypical form of T2D for which IR is absent [24,25]. Additionally, several whole-genome scan studies for T2D susceptibility loci have identified linkage on chromosome 20q12–13 in a region that encompasses the *HNF4A* locus [26–28]. The association between *HNF4A* and T2D has been extensively studied [29]. *HNF4A* genetic variants have been shown to contribute to the risk of T2D in Finnish [30] and Ashkinazi Jewish subjects [31]. These results have been partially replicated in the UK population [32], American Caucasians [33], Amish [34], Danish [35], and French Caucasians [36]. However, other studies did not find associations between *HNF4A* variants and T2D [27,37–39]. Besides, *HNF4A* polymorphisms were found associated with lipid traits, namely levels of high density lipoprotein (HDL) [40–42].

The present study aimed to investigate the relationship between *HNF4A* genetic variants and the presence of metabolic syndrome (MetS) in a pediatric French Canadian population, and to explore their association with metabolic parameters, for instance levels of blood glucose, insulin and lipids.

## METHODS

### Population study

The design and methods of the 1999 Quebec Child and Adolescent Health and Social Survey, a school-based survey of youth aged 9, 13, and 16 years, have previously been reported in detail [43]. On a total of 2,244 DNA samples available [44], we restricted the current analysis to 1,749 children and adolescents of French Canadian origin to reduce the confounding of genetic analyses by population stratification. The study was approved by the Institutional Review Board of Sainte-Justine Hospital and investigations were carried out in accordance with the principles of the Declaration of Helsinki. Written informed consent was obtained from parents/guardians, and written informed assent was obtained from study participants.

### Anthropometry, blood pressure and lipids

Height, weight and blood pressure (BP) were measured according to standardized protocols [43]. Body mass index (BMI) was computed as weight in kilograms divided by height in meters squared. Values of percentile cut-off points used to identify subjects with metabolic risk factors were estimated from the study distributions. Cut-off points were age and sex specific, and BP cut-off points were also height specific, according to the National High Blood Pressure Education Program Working Group on High Blood Pressure in Children and Adolescents [45]. Subjects with BMI ≥ 85th percentile values were categorized as overweight/obese. High triglycerides (TG), insulin, and systolic BP were defined as values ≥ 75th percentile, and low HDL-cholesterol (HDL-C) was defined as values ≤ 25th percentile. Impaired fasting glucose (IFG) was defined as concentrations ≥ 6.1 and < 7.0 mmol/L. No study participant had fasting plasma glucose ≥ 7.0 mmol/L.

Currently, estimating the prevalence of childhood MetS continues to be challenging and controversial and there is no internationally accepted definition of childhood MetS. More than 40 definitions for childhood MetS have so far been proposed and most of them were based on adaptations of adult criteria [1]. Therefore, in the present work, we have based our definition on previously published work from our group, which was useful to assess the clustering of metabolic risk factors and to estimate the prevalence of MetS in a representative sample of youth in the province of Quebec in Canada [46,47]. MetS in our analyses required the presence of obesity and at least two other risk factors among high systolic or diastolic BP, high TG, low HDL-C and IFG [43,48]. In our MetS definition, general obesity was used instead of central obesity since waist circumference data were not available for this study.

### Biochemical analyses

Blood samples were collected in the morning, after an overnight fast. Plasma total cholesterol (TC), HDL-C, TG and glucose concentrations were determined on a Beckman Synchron Cx7 instrument as previously described [43,44]. Apolipoprotein (apo) A-I and apo B were measured by nephelometry (Array Protein System; Beckman). The Friedewald equation was used to calculate low-density lipoprotein-cholesterol (LDL-C). Plasma insulin concentration was determined with the ultrasensitive Access® immunoassay system (Beckman Coulter, Inc.), which has no cross-reactivity with proinsulin or C-peptide. Plasma free fatty acids (FFA) concentrations were quantified by an enzymatic colorimetric method (Wako Chemicals).

### Genotyping

Genomic DNA was prepared from white blood cells using the Puregene® DNA Isolation kit (Gentra Systems, Inc.). The genotyping was carried out as part of a previous study performed on this precise cohort [49]. 24 SNPs with a minor allele frequency > 5% were identified by sequencing the *HNF4A* gene in a French Canadian sample population [49]. The fragments were genotyped using the Luminex xMAP/Autoplex Analyser CS1000 system (Perkin Elmer, Waltham, MA). They were amplified in a single multiplex assay and hybridized to Luminex MicroPlex®—xTAG Microspheres [50] for genotyping using allele-specific primer extension. Amplification and reaction conditions are available upon request. Allele calls were assessed and compiled using the Automatic Luminex Genotyping software [51].

### Statistical Analysis

Statistical analyses were performed with STATA v.10 statistical software (StataCorp LP). Potential genotyping errors were assessed using Chi-square tests, which evaluate the deviation of each SNP from Hardy-Weinberg equilibrium. Subjects were categorized according to their MetS status (yes/no). Between-group allele and genotype frequency distributions were compared by a Chi-square test. Allelic association for individual SNPs was carried out using logistic regression by fitting an additive model. To take the design effect into account, mixed models were used for all analyses of variance and regressions, with genetic markers and other independent variables treated as fixed effects and with clustering between subjects in the same school treated as a random effect. We used mixed logistic regression to examine the association between MetS status and *HNF4A* genotypes. We performed Fisher’s exact test to study the associations for the polymorphism without rare variant. We used mixed ANOVA and mixed linear regression to study the associations between genotypes and metabolic variables. Scheffe’s contrasts were used for posthoc pair comparisons. Insulin, TG, FFA and BMI values were loge transformed for statistical analyses to improve the normality of their distributions. Because we pooled age and sex groups, age- and sex-specific Z scores for BMI, insulin, glucose, TG, LDL-C, HDL-C, apo B and apo A-I were used in linear regression analyses. To standardize a value (i.e., compute its Z score), we subtracted the mean of the corresponding study distribution and divided by the SD. Haplotype analysis was carried out using HAPLOVIEW Software, version 3.11 [52] on the 9 SNPs for which the allelic association was significant or close to significant. Haplotype blocks created using the confidence interval feature. For each block, the haplotype association for each haplotype with MetS was examined by logistic regression. The association with the metabolic markers was evaluated using linear regression and *P* values were estimated.

## RESULTS

### Population Characteristics

The clinical and biochemical characteristics of participants are shown in Table 1. The prevalence of MetS was 11.03%. As expected, youth with MetS displayed significantly higher BMI, systolic and diastolic BP, TC, LDL-C, apo B, TG, FFA, insulin and glucose as well as lower levels of HDL-C and apo A-I than youth without MetS. No differences were detected in age and gender between the two groups. S2 Table indicates the cut-off values according to sex and age for BMI, TG, HDL-C, BP and insulin.

**Table 1:** Characteristics of study participants according to metabolic syndrome status.

Variable	Total (n = 1,749)	MetS^a^		*P* value^b^
		No (n = 1,556)	Yes (n = 193)	
9 year olds, % (n)	31.96 (559)	32.33 (503)	29.02 (56)	0.648
13 year olds, % (n)	30.87 (540)	30.72 (478)	32.12 (62)	-
16 year olds, % (n)	37.16 (650)	36.95 (575)	38.86 (75)	-
Gender: male, % (n)	50.31 (880)	50.39 (784)	49.74 (96)	0.866
BMI^c^ (kg/m^2^)	20.23 ± 4.37	19.30 ± 3.28	28.01 ± 4.55	< 0.00001
Systolic BP (mmHg)	111.88 ± 13.74	110.64 13.00	122.01 ± 15.32	< 0.00001
Diastolic BP (mmHg)	59.34 ± 7.14	58.79 ± 6.95	63.77 ± 7.09	< 0.00001
TC (mmol/L)	4.00 ± 0.75	3.97 ± 0.75	4.20 ± 0.80	< 0.00001
LDL-C (mmol/L)	2.31 ± 0.64	2.28 ± 0.63	2.48 ± 0.67	< 0.00001
Apo B (g/L)	0.66 ± 0.18	0.65 ± 0.17	0.75 ± 0.20	< 0.00001
HDL-C (mmol/L)	1.30 ± 0.25	1.32 ± 0.25	1.13 ± 0.18	< 0.00001
Apo A-I (g/L)	1.19 ± 0.17	1.20 ± 0.17	1.13 ± 0.16	< 0.00001
TG^c^ (mmol/L)	0.87 ± 0.42	0.82 ± 0.36	1.28 ± 0.63	< 0.00001
FFA^c^ (mmol/L)	0.44 ± 0.21	0.43 ± 0.21	0.47 ± 0.20	< 0.0086
Glucose (mmol/L)	5.16 ± 0.38	5.15 ± 0.38	5.26 ± 0.40	< 0.0001
Insulin^c^ (pmol/L)	43.62 ± 30.50	38.71 ± 20.20	83.23 ± 58.24	< 0.00001

Data are expressed as percentage (frequency) or mean ± SD. Apo B, apolipoprotein B; BMI, body mass index; BP, blood pressure; HDL-C, high density lipoprotein-cholesterol; MetS, metabolic syndrome; LDL-C, low density lipoprotein-cholesterol; TC, total cholesterol; TG, triglyceride.

^a^MetS is defined as the presence of obesity (BMI ≥ 85th percentile) in combination with two or more of the following: high systolic BP (≥75th percentile), high diastolic BP (≥75th percentile), high TG (≥75th percentile), low HDL-C (≤ 25th percentile) and impaired fasting glucose (≥ 6.1 mmol/L).

^b^ *P* value for comparisons between groups (MetS- and MetS+).

^c^Untransformed data are presented; log_e_-transformed values were used for statistical comparisons.

### Effect of Polymorphisms on the Risk of Metabolic Syndrome

A total of 1,749 subjects were included for genotyping. Among the 24 SNPs genotyped, two deviated from Hardy-Weinberg equilibrium and were excluded from subsequent analyses (S1 Table). Since rs1884614 was found to be monomorphic, it was also excluded from association analyses. The remaining 21 SNPs were analyzed for association with MetS and results are presented in Table 2. Before correction for multiple testing, there was a significant difference in allele frequencies between MetS- and MetS+ subjects for seven polymorphisms. The minor alleles of two SNPs were associated with an increased risk of MetS (rs1800963, OR: 1.29, *P*<0.025; and rs3212183, OR: 1.38, *P*<0.003), while the minor alleles of five SNPs were associated with a reduced risk of MetS (rs6130608, OR: 0.73, *P*<0.019; rs2425637, OR: 0.74, *P*<0.007; rs3212172, OR: 0.68, *P*<0.030; rs736824, OR: 0.68; *P*<0.001; rs745975, OR: 0.63; *P*<0.003). For two other polymorphisms, the association between the minor allele and the MetS was close to significant (rs6031543, OR: 1.28, *P*<0.092; rs2425639, OR: 0.81, *P*<0.059). Fisher’s exact test was used to analyze the association between rs1884614 and MetS and did not reveal any significant association. However, after correction for multiple comparisons, only the association for rs736824 remained significant (*P*<0.021).

**Table 2:** Association between *HNF4A* polymorphisms and metabolic syndrome: odds ratio.

SNP	Alleles (major/minor)	Minor allele frequency		Odds ratio	95% CI	*P* Value	Corrected *P* value
		Control (n = 1,542)	MetS (n = 207)				
rs4810424	G/C	0.15	0.19	1.17	0.87–1.57	0.300	1.000
rs1884613	C/G	0.14	0.17	1.07	0.79–1.46	0.663	1.000
rs1884614	C/T	0.15	0.18	1.04	0.77–1.41	0.790	1.000
rs6031543	C/G	0.15	0.16	1.28	0.96–1.70	0.092	1.000
rs2144908	G/A	0.14	0.17	1.06	0.78–1.44	0.714	1.000
rs6031550	C/T	0.24	0.23	0.98	0.76–1.26	0.850	1.000
rs6031551	T/C	0.24	0.22	0.96	0.74–1.26	0.787	1.000
rs6031552	C/A	0.23	0.24	1.05	0.82–1.35	0.688	1.000
rs6130716	A/C	0.31	0.29	1.04	0.82–1.32	0.726	1.000
rs6031558	G/C	0.34	0.35	0.99	0.78–1.26	0.942	1.000
rs6130608	T/C	0.27	0.23	0.73	0.56–0.95	0.019	0.399
rs2425637	T/G	0.47	0.45	0.74	0.60–0.92	0.007	0.147
rs2425639	G/A	0.47	0.46	0.81	0.65–1.08	0.059	1.000
rs3212172	A/G	0.15	0.11	0.68	0.48–0.96	0.030	0.630
rs1800963	C/A	0.40	0.45	1.29	1.03–1.61	0.025	0.525
rs2071197	G/A	0.90	0.08	0.95	0.65–1.40	0.805	1.000
rs736824	T/C	0.41	0.36	0.68	0.54–0.86	0.001	0.021
rs745975	C/T	0.22	0.19	0.63	0.47–0.86	0.003	0.063
rs3212183	C/T	0.47	0.49	1.38	1.11–1.72	0.003	0.063
rs1885088	G/A	0.23	0.19	1.12	0.86–1.45	0.408	1.000
rs1800961	C/T	0.03	0.03	1.34	0.71–2.52	0.361	1.000
rs3212195	G/A	0.22	0.18	1.15	0.87–1.53	0.334	1.000

Separate logistic regression models were fit for each SNP adjusting for age, gender and body mass index.

### Effect of Polymorphisms on Metabolic Variables

We studied the effect of *HNF4A* polymorphisms on mean LDL-C, HDL-C, apo A-I, apo B, FFA, TG, glucose and insulin levels. Because we did not detect heterogeneity of effect of *HNF4A* polymorphisms by sex or age, sex and age groups were pooled in subsequent analyses. Regression coefficients were calculated for the interactions between *HNF4A* genotypes and Z-score for blood glucose, insulin, TG, HDL-C, LDL-C, apo A-I and apo B after correction for age, sex and BMI. After correction for multiple testing, one SNP (rs3212172) had minor allele associated with decreased levels of apo B (coefficient: -0.14; *P*<0.001) (Table 3). A negative coefficient suggests decreasing value of the marker for every additional copy of the haplotype. Concomitantly, this allele was also associated with reduced TC (coefficient: -0.09; *P*<0.008) and LDL-C (coefficient: -0.13; *P*<0.008) although these associations did not remain significant after correction for multiple testing.

**Table 3:** Association between rs3212172 and metabolic variables.

SNP	Effect on MetS	Metabolic variables	Adjusted Coefficient	*P* value	Corrected *P* value
rs3212172	↓ risk	TC (mmol/L)	-0.09 ± 0.03	0.008	0.168
		LDL-C (mmol/L)	-0.01 ± 0.05	0.009	0.189
		Apo B (g/L)	-0.14 ± 0.05	0.001	0.021

A negative coefficient suggests decreasing value of the marker for every additional copy of the SNP. The linear mixed model was adjusted for age, gender and body mass index. Apo B, apolipoprotein B; LDL-C, low-density lipoprotein-cholesterol; TC, total cholesterol.

### Haplotype Analyses

**Fig 1:**
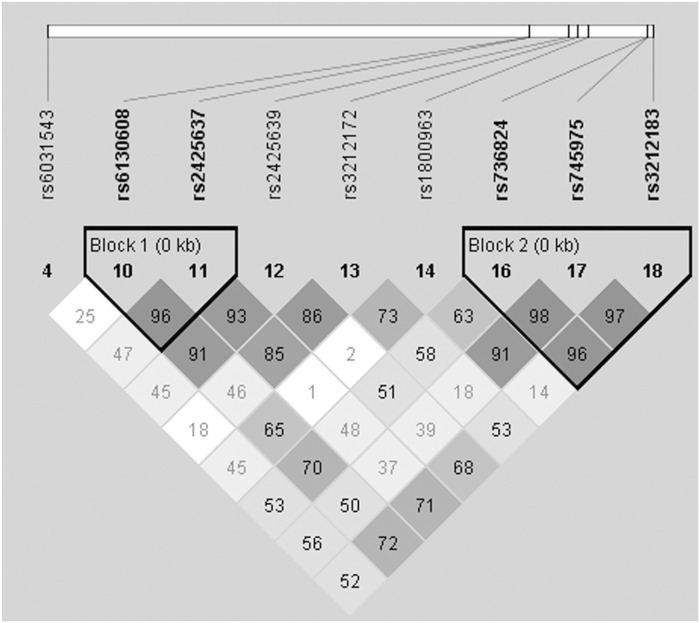
Two major haplotype blocks found in the *HNF4A* gene. Linkage disequilibrium plot in the *HNF4A* region is displayed. Haplotype analysis was carried out on the 9 SNPs for which the single SNP allelic association was significant or close to significant using HAPLOVIEW Software version 3.11.

We then performed linkage disequilibrium (LD) analysis on the 9 SNPs for which the allelic association was significant or close to significant (Fig. 1). This analysis revealed that the SNPs were distributed within two major haplotype blocks: a first block including two SNPs in the region between both promoters (rs6130608, rs2425637) and a second block of three intronic SNPs (rs736824, rs745975, rs3212183). Table 4 shows the frequencies of the identified haplotypes. Haplotype analyses were performed on the SNPs within each block of LD (Table 4). Two haplotypes in the first block (TT and CG) and in the second block (TCC and CTT) were significantly associated with MetS after adjustment for age, sex, BMI, alcohol and cigarette consumption. The association for only one haplotype in each block remained significant after correction for multiple testing (TT, *P*<0.032; CTT, *P*<0.025). On the other hand, these haplotypes were not associated with significant variations in metabolic parameters.

**Table 4:** Association between HNF4α haplotypes and metabolic syndrome.

	Haplotype	Frequency Controls (%)	Frequency MetS (%)	Chi Square	*P* value	Corrected *P* value
***Block 1*** (rs6130608, rs2425637)						
	TT	52.0	59.5	7.67	0.006	0.032*
	CG	26.7	20.3	7.171	0.007	0.052
	TG	20.9	19.9	0.218	0.641	1.000
***Block 2*** (rs736824, rs745975, rs3212183)						
	TCC	45.3	52.4	6.865	0.009	0.074
	CTT	21.7	15.3	8.225	0.004	0.025*
	CCT	18.9	15.2	3.044	0.081	0.353
	TCT	13.4	15.6	1.406	0.236	0.754

*Adjusted value based on permutation methods.

### Study Power

We post-priori estimated the power of the study to detect important associations for the main outcome variable “MetS” that had a frequency of 11.03% in the sample. With the noted range of allele frequencies (15% to 50%), the sample of n = 1,749 participants provided sufficient power (≥ 80%) to detect odds ratio (OR) ≥ 1.55 (or ≤ 0.65) after correcting for multiple comparisons (alpha set at 0.002 for correcting for ~25 SNP associations). For lower OR (in the range 1.2 to 1.5), the study did not have adequate power. For OR of 1.3, the study power ranged from 11% to 28%, while for OR of 1.4 it ranged from 26% to 53% and for OR of 1.5 it ranged from 46% to 77% for an allele frequency range between 15% and 45%. As for the metabolic variables, based on the distribution of the mean (SD) of the levels in the population, estimated β-coefficient and correcting for multiple comparisons (alpha = 0.002), power of the study ranged from 5% to 100% depending on the metabolite and the allele frequency of the SNP.

## DISCUSSION

**Fig 2:**
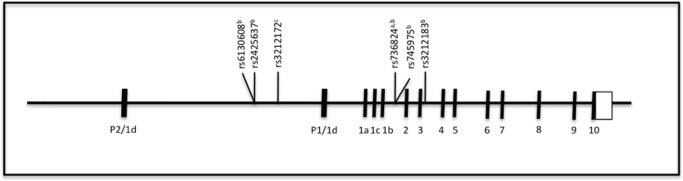
Schematic illustration of the *HNF4A* SNPs found associated with metabolic syndrome or metabolic parameters. Relative position of SNPs within the *HNF4A* locus. ^a^SNP associated with the metabolic syndrome; ^b^haplotype associated with metabolic syndrome; ^c^SNP associated with apo B levels.

In the present study, we aimed to evaluate the association between *HNF4A* genetic variants and MetS in French Canadian children and adolescents. Our analyses revealed that, after correction for multiple testing, one SNP (rs736824) and two haplotypes (P1 promoter haplotype rs6130608-rs2425637 and intronic haplotype rs736824-rs745975-rs3212183) were significantly associated with the risk of MetS (Fig. 2). Additionally, another significant association was found between rs3212172 and apo B levels (Fig. 2). To our knowledge, this is the first study exploring the relation between *HNF4A* genetic variants, MetS and metabolic variables in a pediatric population. Single SNP analysis revealed that the presence of the minor allele C of the intronic SNP rs736824 (intron 1A/1B-2) was associated with a 0.68 fold reduced risk of MetS. While this SNP was not found associated with T2D in American Caucasians [33] or with the conversion to T2D in the STOP-NIDDM trial [53], it was independently associated with fasting glucose levels in North Indians of Indo-European control subjects [54]. Moreover, an intronic haplotype (rs736824-rs745975-rs3212183) containing the rs736824 C allele was also protective for MetS. Accordingly, rs3212183 was modestly associated with T2D (OR: 1.34) in Pima Indians [55] and the protective effect of the T allele was confirmed in a meta-analysis (OR: 0.843) carried out on 4 studies [56]. Also, a haplotype containing the polymorphism rs745975 (rs745975-s2425640) has been associated with TG and glucose levels in Mexicans [57] but was never found independently associated with T2D or metabolic parameters in the literature.

Among the SNPs identified in our study, rs2425637, which is part of the P1 promoter haplotype associated with risk of MetS, has been the most reported in the literature. It was found associated with T2D in Finnish, Ashkenazi and French Caucasian populations [58,59], but not with conversion to T2D in the STOP-NIDDM trial [53]. In a meta-analysis, a haplotype containing rs2425637 was not significantly associated with T2D, except for a marginal effect in Scandinavians [56].

The minor allele G of the P1 promoter SNP rs3212172 showed overall cardioprotective effects since it was linked to decreased risk of MetS and lower levels of TC, LDL-C and apo B, with only the latest remaining significant after correction for multiple testing. To our knowledge, no association has previously reported in the literature for that polymorphism. Since HNF4α is known to regulate apo B gene expression [60,61], the functional impact of this particular SNP would be particularly interesting to explore. In fact, it has been demonstrated that MODYI patients have lower levels of very-low density lipoprotein-C and LDL-C than controls, which was attributed, at least in part, to a reduced transactivation activity of HNF4α for the acyl-coenzyme A: cholesterol acyltransferases 2 promoter [62]. Although the association between TG levels and MODY1 (HNF4A Q268X mutation) has previously been demonstrated [63], the correlation does not hold true when assessed with *HNF4A* common SNPs in our French Canadian population.

Interestingly, the *HNF4A* genetic variants identified in our study are located in P1 promoter and intronic regions; none of the P2 promoter SNPs was found associated with MetS or with metabolic parameters. Conversely, in the literature, attention has been mostly paid to P2 promoter SNPs. Evidence for association between SNPs in the beta-cell P2 promoter region of *HNF4A* was recognized in Finnish [30] and Ashkenazi [31,64] populations, with data suggesting that *HNF4A* P2 SNPs (or variants in strong linkage disequilibrium with them) contribute to the linkage signal on chromosome 20q [30,31]. Yet, association with *HNF4A* promoter SNPs has been replicated in some [65] but not all [66,67] populations tested. Hence, there was evidence for association with SNPs or haplotypes in the *HNF4A* region other than the P2 SNPs [68,69]. Moreover, a meta-analysis showed that P2 promoter SNPs were associated with T2D only in Scandinavians [56]. Recently, P2 promoter SNPs have been associated with insulin resistance and BMI in adult subjects [70], but the study was performed on a small sample size of 160 subjects. Data obtained in our study support the lack of association between P2 promoter variants and metabolic parameters in children.

Functional studies have initially reported that the P2 promoter drives transcription in β-cells and that the P1 promoter drives transcription in extra-pancreatic cells, such as liver cells [19,21]. However, studies have previously linked P1 promoter polymorphisms to T2D and, along with our study, suggest important contribution for P1-driven genes in insulin resistance, glucose tolerance and MetS development.

The fact that different SNPs in the *HNF4A* region are associated with diabetes in different populations suggests that none of these alleles themselves are causative functional variants but that they may be in linkage disequilibrium with a nearby functioning allele. Alternatively, some of these alleles may be causative, but allelic heterogeneity across populations may make their identification difficult. Moreover, it has been suggested that HNF4α can be constitutively bound to fatty acids [71] and it can bind to linoleic acid in a reversible fashion [72]. HNF4α was revealed as important for hepatic response to changes in nutritional status [73]. Hence, diverge results in association studies might be explained by the dietary influence that might play a role and dilute the genetic impact to a variable extent depending on the study population [29].

As mentioned before, the definition of overweight/obesity in children (BMI ≥ 85th percentile) used herein was based on our previous publications performed on a representative Canadian population [46,47]. This definition also corresponds to the one proposed by the Center for Disease Control and Prevention (CDC). According to their charts, the CDC defines overweight as a BMI above the 85^th^ percentile of the reference population and obesity as a BMI above the 95^th^ percentile [74]. Moreover, the World Health Organization (WHO) system defines overweight as a BMI > 1 SD and obesity as a BMI > 2 SD from the mean of the WHO reference population [75]. The WHO reference BMI-for-age curves at 19 years closely coincides with adult overweight (BMI = 25.0 kg/m^2^) at +1 SD and adult obesity (BMI = 30.0 kg/m^2^) at +2 SD. It was found that these obesity and overweight cut-off values identified children with higher metabolic and vascular risk [76]. According to our reference population, the 85^th^ percentile corresponds in a BMI of 20.04, 23.85 and 26.45 kg/m^2^ for boys of 9, 13 and 16 years old, respectively, and of 20.51, 26.01 and 26.25 kg/m^2^ for girls of 9, 13 and 16 years old, respectively (S2 Table). According to the WHO charts, these values correspond in boys to +2 SD for the 9-year-old age group and +1.5 SD for the 13- and the 16-year-old groups, and in girls to +2 SD for the 9- and 13-year-old age groups and +1.5 SD for the 16-year-old group.

Despite the continued use of the MetS concept, a number of ongoing issues surround the MetS notion and its application to children. Children, unlike the adults for whom the MetS concept was originally developed, reside in vastly different stages of growth, development and pubertal status, thereby questioning whether such variability can be accommodated by a single MetS definition [77]. Another difficulty is the fact that reference values for some MetS components, such as waist circumference, exist for only some populations and that there remains disagreement over how to measure waist circumference in children [77]. Also, the lack of reference values in some populations for blood pressure or HDL-C level render cross-cultural comparisons problematic [78]. On the other hand, the use of dichotomous (normal vs. abnormal) variable categories is also debated. Strict cut-off points are difficult to apply in the pediatric population given the well-known fluctuations associated with growth and puberty [77]. For these reasons and based on previous studies from our group [46,47] we have decided to identify a sub-group of children in our population who are more at risk of cardiovascular complications and we have identified that group as MetS+. Importantly, the definition used in this manuscript identified 11.03% of the population with MetS, which corresponds to what we have found in a previous investigation [46]. As a matter of fact, several definitions of the MetS have been compared using this specific population and the overall prevalence of MetS was ranging between 11.5% and 14.0% according to the stringency of the definition [46].

This study presents a certain number of limitations. First, because waist circumference values were not available for this study, the International Diabetes Federation diagnostic criteria for MetS in children and adolescent could not have been used. Also, data available in this study did not make possible the analysis between HNF4A polymorphisms and previously reported lipid abnormalities in MODY1 such as apo A-II, apo C-III and lipoprotein (a)

In conclusion, this study, the first exploring the relation between *HNF4A* genetic variants, MetS and metabolic variables in a pediatric cohort, supports the hypothesis that *HNF4A* P1 promoter and intronic polymorphisms play a role in predisposing to T2D and could represent an early marker for the risk of developing the disease.

## Supporting Information

S1 Table Genotyped SNPs and Hardy-Weinberg equilibrium test. Among the 24 SNPs genotyped, two deviated from Hardy-Weinberg equilibrium and were excluded from subsequent analyses. HWE, Hardy-Weinberg equilibrium. *SNPs with a significant HWE test were excluded for further analyses. **SNP with no rare homozygote was excluded for association analyses.
(PDF)

S2 Table Cut points used to define risk factors by age and sex. The cut points correspond to the 85^th^ percentile of the study population for BMI, the 75^th^ percentile for triglycerides, insulin, systolic BP and diastolic BP and the 25^th^ percentile for HDL-cholesterol. BMI, body mass index; BP, blood pressure.
(PDF)
