# Supplementary material for: Hepatocyte Nuclear Factor 4 Alpha Polymorphisms and the Metabolic Syndrome in French-Canadian Youth
Source: PLoS One. 2015 Feb 11;10(2):e0117238. doi: 10.1371/journal.pone.0117238 (PMC4325000; doi:10.1371/journal.pone.0117238)
Supplement: S1 Table — HWE, Hardy-Weinberg equilibrium. *SNPs with a significant HWE test were excluded for further analyses. **SNP with no rare homozygote was excluded for association analyses. (PDF) [file pone.0117238.s001.pdf]

S1 Table: Genotyped SNPs and Hardy-Weinberg equilibrium test

SNP	Position	HWE test *P* value
**rs4810424**	Promoter 2	0.1655
**rs1884613**	Promoter 2	0.6920
**rs1884614**	Promoter 2	**no rare homozygote****
**rs6031543**	Promoter 2	0.9245
**rs2144908**	Intron 1A-1D	0.2945
**rs6031550**	Intron 1A	0.6862
**rs6031551**	Intron 1A	0.5303
**rs6031552**	Intron 1A	1.0000
**rs6130716**	Intron 1A	0.0720
**rs6031558**	Intron 1A	0.1798
**rs6130608**	Promoter 1	0.5189
**rs2425637**	Promoter 1	0.4675
**rs2425639**	Promoter 1	0.9232
**rs3212172**	Promoter 1	1.0000
**rs1800963**	Promoter 1	0.1923
**rs2071197**	Promoter 1	0.8835
**rs736824**	Intron 1	0.6889
**rs745975**	Intron 1	0.7714
**rs3212183**	Intron 2	0.1299
**rs1885088**	Intron 3	0.2085
**rs1800961**	Exon 4	1.0000
**rs3212195**	Intron 4	0.8727
**rs3212198**	Intron 4	**0.0126***
**rs3818247**	Intron 9	**0.0032***

HWE, Hardy-Weinberg equilibrium. *SNPs with a significant HWE test were excluded for further analyses. **SNP with no rare homozygote was excluded for association analyses.
