# Supplementary material for: Hepatocyte Nuclear Factor 4 Alpha Polymorphisms and the Metabolic Syndrome in French-Canadian Youth
Source: PLoS One. 2015 Feb 11;10(2):e0117238. doi: 10.1371/journal.pone.0117238 (PMC4325000; doi:10.1371/journal.pone.0117238)
Supplement: S2 Table — BMI, body mass index; BP, blood pressure. (PDF) [file pone.0117238.s002.pdf]

S2 Table: Cut points used to define risk factors by age and sex

Sex	Age (y)	BMI (kg/m^2)	Triglycerides (mmol/L)	HDL-C (mmol/L)	Systolic BP (mmHg)	Diastolic BP (mmHg)	Insulin (pmol/L)
**Boys**	**9**	20.04	0.83	1.22	109	60	33.73
	**13**	23.85	1.01	1.11	121	63	56.16
	**16**	26.45	1.08	1.00	134	66	49.19
**Girls**	**9**	20.51	0.96	1.21	108	61	39.03
	**13**	26.01	1.08	1.13	118	65	71.26
	**16**	26.25	1.23	1.13	121	67	61.09

These cut points correspond to the 85th percentile of the study population for body mass index (BMI), the 75th percentile for triglycerides, insulin, systolic blood pressure (BP) and diastolic BP and the 25th percentile for HDL-cholesterol.
